# Achieving pH control in microalgal cultures through fed-batch addition of stoichiometrically-balanced growth media

**DOI:** 10.1186/1472-6750-13-39

**Published:** 2013-05-07

**Authors:** Megerle L Scherholz, Wayne R Curtis

**Affiliations:** 1 Department of Chemical Engineering, Pennsylvania State University, University Park, PA, 16802, USA

**Keywords:** Microalgae, Nitrogen metabolism, pH control, Stoichiometry, High density, *Chlorella vulgaris*, *Chlamydomonas reinhardtii*

## Abstract

**Background:**

Lack of accounting for proton uptake and secretion has confounded interpretation of the stoichiometry of photosynthetic growth of algae. This is also problematic for achieving growth of microalgae to high cell concentrations which is necessary to improve productivity and the economic feasibility of commercial-scale chemical production systems. Since microalgae are capable of consuming both nitrate and ammonium, this represents an opportunity to balance culture pH based on a nitrogen feeding strategy that does not utilize gas-phase CO_2_ buffering. Stoichiometry suggests that approximately 36 weight%N-NH_4_^+^ (balance nitrogen as NO_3_^-^) would minimize the proton imbalance and permit high-density photoautotrophic growth as it does in higher plant tissue culture. However, algal media almost exclusively utilize nitrate, and ammonium is often viewed as ‘toxic’ to algae.

**Results:**

The microalgae *Chlorella vulgaris* and *Chlamydomonas reinhardtii* exclusively utilize ammonium when both ammonium and nitrate are provided during growth on excess CO_2_. The resulting proton imbalance from preferential ammonium utilization causes the pH to drop too low to sustain further growth when ammonium was only 9% of the total nitrogen (0.027 gN-NH_4_^+^/L). However, providing smaller amounts of ammonium sequentially in the presence of nitrate maintained the pH of a *Chlorella vulgaris* culture for improved growth on 0.3 gN/L to 5 gDW/L under 5% CO_2_ gas-phase supplementation. Bioreactor pH dynamics are shown to be predictable based on simple nitrogen assimilation as long as there is sufficient CO_2_ availability.

**Conclusions:**

This work provides both a media formulation and a feeding strategy with a focus on nitrogen metabolism and regulation to support high-density algal culture without buffering. The instability in culture pH that is observed in microalgal cultures in the absence of buffers can be overcome through alternating utilization of ammonium and nitrate. Despite the highly regulated array of nitrogen transporters, providing a nitrogen source with a balanced degree of reduction minimizes pH fluctuations. Understanding and accommodating the behavior of nitrogen utilization in microalgae is key to avoiding ‘culture crash’ and reliance on gas phase CO_2_ buffering, which becomes both ineffective and cost-prohibitive for commercial-scale algal culture.

## Background

Algae-based biofuels have been gaining attention as a potential production platform for renewable fuel and biochemicals. Algal systems offer advantages over terrestrial plant sources, such as higher productivity, increased oils, avoidance of food-for-fuel, and the potential for using both wastewater and saltwater [1,2]. However, an improved understanding of nutrient utilization in algal cultures is needed to develop the high density culturing methods that are required to achieve economic feasibility of these systems. Increasing reactor productivity through application of high-density cell concentration reduces downstream harvesting costs. The work in this article aims to achieve this goal by maintaining culture pH through the use of stoichiometrically- balanced growth media.

High density algal culture requires the development of media which provides high inorganic salt concentrations while avoiding the accumulation of inhibitory levels of counter-ions [3]. Metabolic flux analysis has been used in an effort to characterize relative rates of consumption in cellular processes, but is only as effective as our limited understanding of algal metabolism [4]. Another approach to formulate growth media is through an examination of the biomass composition [5]. This method is effective for optimization of key inorganic components, but does not address the coupling of nitrogen utilization with the energy balance as reflected in the oxygen and hydrogen components of the biomass. A better understanding of the overall stoichiometry and energetics of growth including water splitting to provide reducing power is required to achieve the goal of ultra-high density algal cultivation.

Photosynthetic growth presents a major challenge to stoichiometry that is rarely appreciated – even by those who have studied it for space life support systems! The presumed photosynthesis growth equation allows for the direct calculation of the stoichiometric coefficients in terms of biomass composition (x, y, and z) once a nitrogen source $\left(N_{i}\right)$ is specified (Equation 1). Table 1 is a compilation of the stoichiometric coefficients on potential nitrogen sources for photoautotrophic growth under the base condition of no extracellular metabolite excretion (A more complete listing is available in Additional file 1).

(1) $$\alpha_{i}CO_{2}\;+\;\psi_{i}N_{i}+\delta_{i}H_{2}O\overset{\mathrm{hv}}{\to }CH_{x}N_{y}O_{z}+\lambda_{i}O_{2}$$

**Table 1:** Algebraic expression of stoichiometric coefficients for common nitrogen sources

**Nitrogen source**	**Stoichiometric coefficients**			
	***α***	***Ψ***	***δ***	***λ***
N_2_	1	$\frac{y}{2}$	$\frac{x}{2}$	$1+\frac{x}{4}-\frac{z}{2}$
NH_4_^+^	1	*y*	$\frac{x}{2}-2y$	$1+\frac{x}{4}-y-\frac{z}{2}$
NO_3_^−^	1	*y*	$\frac{x}{2}$	$1+\frac{x}{4}+\frac{3y}{2}-\frac{z}{2}$
CO(NH_2_)_2_	$1-\frac{y}{2}$	$\frac{y}{2}$	$\frac{x}{2}-y$	$1+\frac{x}{4}-\frac{3y}{4}-\frac{z}{2}$

The stoichiometric coefficients were determined algebraically from Equation 1 and are given in terms of biomass composition (x, y, z) for various nitrogen sources when extracellular metabolites are not considered.

This array of stoichiometric solutions for photoautotrophic growth emphasizes the need for an additional consideration in the overall energy balance. Unlike heterotrophic or autotrophic growth where the energy production reaction (i.e. combustion) can be decoupled from biomass formation, the same cannot be done for photoautotrophic growth. Decoupling of energy and biomass production to balance stoichiometry is achieved by specifying a heterotrophic biomass yield on a particular substrate for a given organism. While additional energy is available for photosynthetic growth through the splitting of water and reduction of electron carriers $\left(I\right)$ such as ferredoxin or NADP^+^ as shown in Equation 2, there is no outlet for these “waste” hydrogen atoms in the basic photosynthetic growth equations.

(2) $$2H_{2}O\overset{\mathrm{hv}}{\to }O_{2}+4H^{+}\left(I\right)$$

According to growth as constrained by Equation 1, these “waste” hydrogen atoms would have to be absorbed into the biomass, requiring changes in biomass composition. However, the composition of biomass is rather constant across organisms, which illustrates the need for modification of the basic growth equation to account for this proton imbalance or to decouple the energy balance from growth as shown in Equation 3.

(3) $$\alpha_{i}CO_{2}+\psi_{i}N_{i}+\delta_{i}H_{2}O\;\overset{\mathrm{h\upsilon}}{\to }CH_{x}N_{y}O_{z}+\lambda_{i}O_{2}+\phi_{i}H^{+}$$

The nature of ϕ_i_ for different nitrogen sources and its relation to energetics will be the focus of subsequent study. In this paper our objective is to design media to facilitate pH control (ϕ_i_ ≈ 0) using a combination of nitrogen sources with different degrees of reduction. The issue of pH control is implicitly appreciated in typical culture methods for algae. Ammonium is rarely used for growth because of its associated ‘toxicity’

$$\left(\phi_{NH_{4}^{+}}\;>0\right)$$

[6-11]. Growth on nitrate is linked to a rise in pH $\left(\phi_{NO_{3}^{-}}<0,a\;\mathrm{net}\;\mathrm{uptake}\;\mathrm{of}\;\mathrm{protons}\right)$ and is routinely used in combination with elevated CO_2_ to suppress pH changes through buffering associated with bicarbonate equilibrium. High gas-phase CO_2_ supplementation is comparable to the direct use of bicarbonate salts as buffers. In addition to pH buffering, the bicarbonate equilibrium system affects CO_2_ transport and the kinetics of growth. Elevated pH provides a greater driving force for CO_2_ transport due to the shift in carbon dissolution in water as shown in Equation 4. However, a neutral or acidic pH is more favorable for carbon fixing microalgae at the cellular level because bicarbonate and dissolved CO_2_ are preferred over carbonate [12-14].

(4) $$CO_{2}{}_{\left(g\right)}↔CO_{2}{}_{\left(\mathrm{aq}\right)}+H_{2}O↔H_{2}CO_{3}↔H^{+}+\mathrm{HC}O_{3}^{-}↔H^{+}+CO_{3}^{2-}$$

While elevated CO_2_ provides for buffering of pH, this excess CO_2_ presents severe limitations to achieving high yield of CO_2_ use in a photosynthetic system. The unused CO_2_ that is not taken up by cells is ultimately released into the atmosphere. Providing elevated CO_2_ is expensive, unsafe, not sustainable, and difficult to implement in large-scale algal culture systems. To improve both the economic feasibility of commercial scale systems and reduce greenhouse gas emissions, it is desirable to maximize CO_2_ yield, which will require reducing the gaseous CO_2_ supplementation level. Therefore, typical pH control achieved through nitrate metabolism and elevated CO_2_ is not feasible. Buffers and acid/base addition are alternative pH control methods for bench-scale reactors, but result in accumulation of counter ions which can contribute to ‘culture crash’ when operating continuously at ultra-high density (unpublished observation). As a result, we have developed a new pH control strategy based on the stoichiometry of growth to allow for maintaining pH at the commercial-scale.

### Defining a balanced NH_4_^+^/NO_3_^-^ media

A medium containing both ammonium and nitrate provides the opportunity to balance external pH (ϕ_i_ = 0) for prolonged growth. This approach has been empirically developed in plant tissue culture, where growth to high densities is achievable as plant cells selectively consume ammonium and nitrate to balance their external pH [8,15-18]. In keeping with the long-term goal of media that supports ultra-high density algal cultivation, the ability to provide a significant amount of nitrogen as NH_4_NO_3_ has the added advantage of avoiding inhibitory accumulation of counter-ions. The stoichiometric growth equation for co-provision of ammonium and nitrate is written as shown in Equation 5, where Δ is the fraction of nitrogen present in the form of ammonium given as Δ = [*N* − *NH*_4_^+^]/([*N* − *NO*_3_^−^] + [*N* − *NH*_4_^+^]). Since the ammonium degree of reduction is large, Δ is expected to be less than 0.5.

(5) $$\begin{array}{l} \mathrm{\alpha C}O_{2}+\Delta \psi NH_{4}^{+}+\left(1-\Delta \right)\psi NO_{3}^{-}\\ \;+\delta H_{2}O\;\overset{\mathrm{h\upsilon}}{\to }CH_{x}N_{y}O_{z}+\lambda O_{2}+\phi H^{+} \end{array}$$

While an exhaustive review of literature revealed extensive discussion of inconsistences and problems attempting to resolve photosynthetic efficiency [19-22], there are no reports where the mass balance provided by Equation 3 has accurately accounted for the proton imbalance. Instead, the most detailed studies of algal growth mass balance have utilized urea [23,24], which has empirically been observed to achieve a balanced pH during continuous growth (ϕ_*urea*_ ≈  0). The balance in the degree of reduction achieved for photosynthetic growth on urea represents an energetic constraint we wish to reproduce in a new algal growth medium through the selection of an appropriate ammonium fraction. This rationale was the basis for equating the amount of carbon dioxide that is fixed relative to the amount of water that is split (*α*/*δ*) for these two media alternatives. By setting ${\left(\frac{\alpha }{\delta }\right)}_{\mathrm{urea}}$ equal to ${\left(\frac{\alpha }{\delta }\right)}_{\left(NH_{4}^{+}:NO_{3}^{-}\right)}$, Δ can be expressed in terms of the biomass composition as given in Equation 6. Details of the algebraic stoichiometric analysis are provided in Additional file 2.

(6) $$\Delta =\frac{4-x}{4\left(2-y\right)}$$

Excluding storage compounds such as lipids and carbohydrates, the composition of biomass is nearly constant across organisms. The stoichiometry of biomass growth has often relied on the detailed compositional analysis of *E. coli*, CH_1.776_ N_0.165_O_0.495_[20,25], which corresponds to 9.6% nitrogen by mass (10.4 g biomass/gN). This biomass composition gives a 30% ammonium composition (Δ = 0.3) in our mixed nitrogen source media design (Equation 6). The biomass composition of *Chlorella* has been reported as CH_1.73_ N_0.067_O_0.327_ (4.7% N by mass) and for *Chlamydomonas* as CH_1.82_ N_0.103_O_0.594_ (5.8% N by mass) [26-28], which both give Δ = 29%. This value of ammonium-nitrogen is consistent with the composition of MS media [29] for plant tissue culture (Δ_MS_ = 0.355), which have been arrived at empirically for heterotrophic growth without pH control *ϕ*_*MS*_ ≈ 0.

Keeping in mind that the goal to produce biofuels involves the accumulation of a product (i.e. intracellular or extracellular lipid) that has a very high carbon to oxygen ratio. This product can be included in the stoichiometric growth equation as shown in Equation 7, where β is an experimentally determined yield for the product such as%lipid $\;\left(\frac{\beta }{1+\beta }\times 100\%\right)$ after conversion from a mass to molar basis.

(7) $$\begin{array}{l} \mathrm{\alpha C}O_{2}+\Delta_{p}\mathrm{\psi N}H_{4}^{+}+\left(1-\Delta_{p}\right)\mathrm{\psi N}O_{3}^{-}+\delta H_{2}O\;\overset{\mathrm{h\upsilon}}{\to }CH_{x}N_{y}O_{z}\\ \;+\mathrm{\beta C}H_{p}N_{q}O_{r}+\lambda O_{2}+\phi_{p}H^{+} \end{array}$$

For a fuel molecule, the carbon to oxygen ratio will be high such that r « z and nitrogen will not be present (q = 0). Since the degree of reduction of a fuel product is greater than that of the biomass, there will be an increased demand for a reduced nitrogen source (Δ_fuel_ > Δ_biomass_) with the added energetic advantage of feeding more ammonium relative to nitrate [30,31]. Therefore, the nitrogen ratio that will achieve a proton balance will be dependent upon the level and composition of products formed. An ammonium level of 36% of the total nitrogen (36%N-NH_4_^+^) was chosen as the base media composition for pH-balanced algal growth. In the results below, the observation of differential nitrogen uptake will be shown to dramatically affect the dynamics of pH. Therefore, further refinement of the proton balance involves controlling the dynamics of nitrogen availability in addition to the stoichiometric composition. The work that follows demonstrates how fed-batch addition of our stoichiometric media can be used to overcome the pH instability that results from the inability of microalgal cultures to selectively consume either ammonium or nitrate.

## Results and discussion

### Proton imbalance in microalgal cultures is caused by preferential ammonium uptake

**Figure 1:**
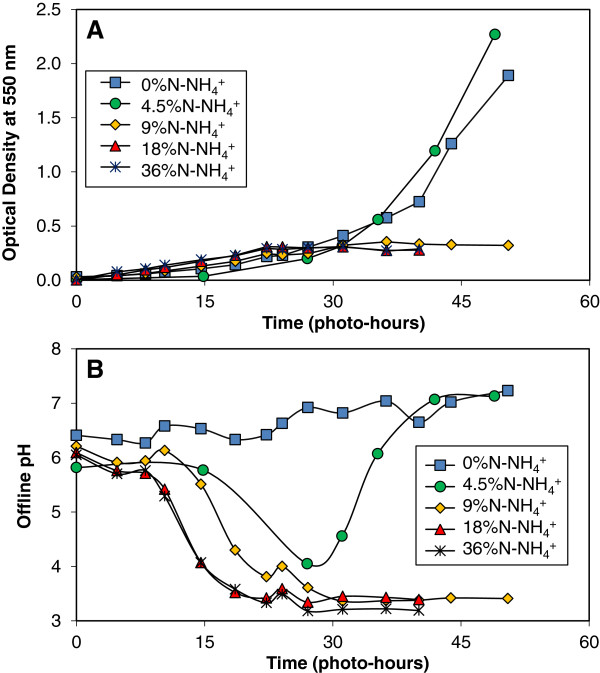
**pH “toxicity” from nitrogen metabolism in *Chlorella vulgaris*.** Photoautotrophic *Chlorella vulgaris* cultures were grown in shake flasks with 5% CO_2_ (v/v) in air on 0.3 gN/L with 0 to 36% as ammonium (0 to 0.108 gN-NH_4_^+^/L) and the remaining as nitrate (0.3 to 0.192 gN-NO_3_^-^/L). The highest ammonium concentration corresponded to the nitrogen composition of our stoichiometrically-balanced photoautotrophic growth media. The optical density was measured at 550-nm to monitor culture growth as a proxy for dry weight (**A**). Offline pH samples were taken at 3-hr intervals, degassed, and measured in anticipation of a proton imbalance due to nitrogen metabolism (**B**).

*Chlorella vulgaris* cultures were grown in 1.5-L loop air-lift photobioreactors (light path-length limited to 0.75-in.) under 5% CO_2_ (v/v) in air with 0.3 gN/L provided as 0-36% from ammonium (0 to 0.108 gN-NH_4_^+^/L) and the balance as nitrate. The highest ammonium concentration corresponded to our growth medium designed to be stoichiometrically-balanced for un-buffered pH control (Background). Cultures provided with 0 and 4.5% nitrogen from ammonium displayed significant growth as shown in Figure 1A, whereas higher levels of ammonium caused growth to stop in early log phase. As anticipated, the culture grown on nitrate-only demonstrated a steady rise in pH over the growth period as a net influx of protons was required to support metabolism (Figure 1B). In contrast, cultures grown on the ammonium levels of 9% or more fell below a pH of 4 and stopped growing. Even the lowest level of ammonium addition at 4.5%N-NH_4_^+^ resulted in a dramatic decrease to pH = 4 during the growth phase followed by subsequent pH rise indicating sequential ammonium and nitrate metabolism. These observations strongly suggest the inability of *Chlorella vulgaris* to utilize nitrate to balance pH and sustain growth when ammonium was present.

**Figure 2:**
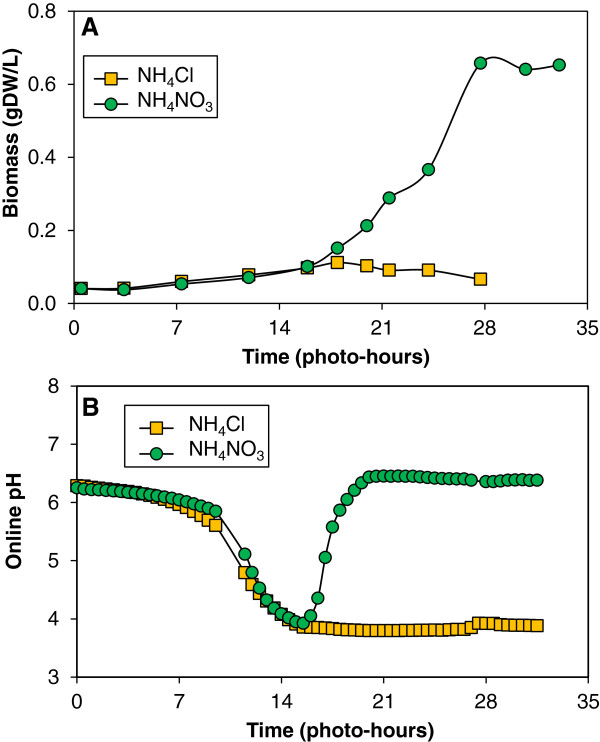
**Inhibition of nitrate assimilation by ammonium in *Chlorella vulgaris.*** Photoautotrophic *Chlorella vulgaris* cultures were grown in 1.5-L loop air-lift photobioreactors on 0.0135 gN-NH_4_^+^/L with chloride and nitrate as the counter-ions. The reactor was supplemented with 5% CO_2_ (v/v) in air. The optical density was measured at 550-nm at 3 to 4-hr intervals and was converted to biomass density using a ratio of 0.52 gDW/L/OD_550_ (**A**). The pH was measured continuously online and averaged at 30-min intervals to track the proton imbalance due to nitrogen utilization (**B**).

To further substantiate that the observed pH swing in the surviving culture was due to ammonium metabolism followed by nitrate, a second batch experiment was executed to compare the pH drop in *Chlorella vulgaris* cultures given ammonium with either nitrate or chloride as the counter-ion (Figure 2). Continuous pH monitoring was implemented within the 1.5-L loop air-lift photobioreactors supplemented with 5% CO_2_ (v/v) in air. Both reactors contained the same initial level of ammonium, 0.0135 gN-NH_4_^+^/L (4.5% N as NH_4_^+^) and experienced nearly identical pH drop during the initial growth phase. The culture grown on NH_4_Cl ceased growth whereas the ammonium nitrate culture displayed a subsequent recovery of pH and continued growth. This observation is consistent with nearly exclusive utilization of ammonium over nitrate and corroborates previous reports that nitrate utilization is inhibited in the presence of ammonium [30-32]. It is notable that *Chlorella* was capable of surviving a pH swing to below 4 where the bicarbonate equilibrium is shifted almost entirely to aqueous dissolved CO_2_. Assuming exclusive consumption of ammonium during the initial drop in pH and not considering buffering, the change in proton concentration during ammonium consumption for both cultures was 0.136 ± 0.015 mol H^+^/mol N-NH_4_^+^. It is interesting to note that the apparent yields on nitrogen are much lower for ammonium as compared to nitrate: 7.13 ± 0.18 gDW/gN-NH_4_^+^ and 14.52 ± 0.61 gDW/gN-NO_3_^-^, respectively. The value of nitrogen yield for generic biomass (CH_1.78_ N_0.165_O_0.495_) is 10.4 gDW/gN; the observed lower biomass yield on ammonium would be consistent with accumulation within the cell in excess of the minimum requirement for biomass formation.

**Figure 3:**
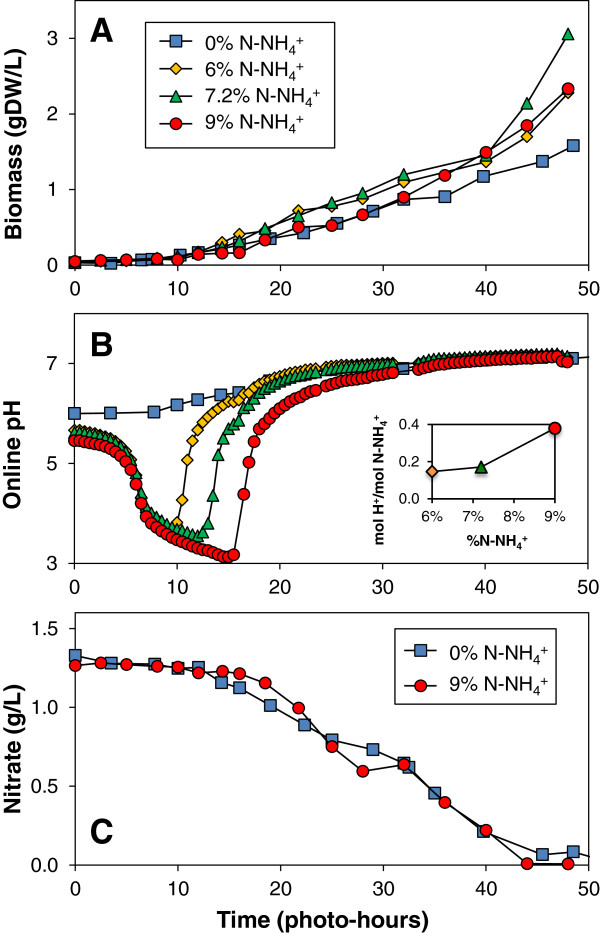
**pH instability from nitrogen metabolism in *Chlamydomonas reinhardtii*.** Photoautotrophic *Chlamydomonas reinhardtii* cultures were grown in 1.5-L loop air-lift photobioreactors on 5% CO_2_ (v/v) in air and 0-9% nitrogen from ammonium (0 to 0.027 gN-NH_4_^+^/L) with the balance nitrogen as nitrate. The optical density was measured at 550-nm to monitor culture growth and converted to biomass density using the conversion factor of 0.52 gDW/L/OD_550_ determined experimentally (**A**). The culture pH was monitored online continuously with samples averaged every 5 minutes to monitor nitrogen utilization (**B**). The nitrate concentration was measured offline using an ion selective electrode (**C**).

Similar batch experiments were executed with *Chlamydomonas reinhardtii* to determine how this ‘model’ microalga responded to mixed nitrogen sources. Photoautotrophic cultures were grown in 1.5-L loop airlift photobioreactors under 5% CO_2_ (v/v) in air on media with 0.3 gN/L with ammonium provided as 0-9% of the nitrogen (0 to 0.027 gN-NH_4_^+^/L) and the balance as nitrate. All cultures continued to grow during the 50 photo-hour growth period as shown in Figure 3A. The minimum pH observed in the *Chlamydomonas* cultures was proportional to the initial ammonium level provided. *Chlamydomonas reinhardtii* grew despite reaching a pH as low as 3 (Figure 3B), whereas *Chlorella vulgaris* did not sustain growth below a pH of 3.75. As further evidence for the preferential uptake of nitrate in the presence of ammonium, the nitrate concentration measured by an ion-selective probe was initially constant until the minimum pH was reached (Figure 3C). This provides more conclusive evidence that alga cells did not take up nitrate in the presence of ammonium. The observed biomass yields on nitrogen for *Chlamydomonas reinhardtii* cultures during the respective nitrogen assimilation phases were 4.91 ± 0.16 gDW/gN-NH_4_^+^ and 6.10 ± 0.13 gDW/gN-NO_3_^-^ respectively. The lower biomass yields on nitrogen for *Chlamydomonas* are consistent with a reduced accumulation of fatty acid lipids as compared to *Chlorella*. The low biomass yield of *Chlamydomonas* indicates an accumulation of nitrogen within the cells to over 16.4% by mass, compared to 9.6% for generic biomass.

Towards achieving pH control using nitrogen feeding, proton secretion in conjunction with ammonium metabolism can be calculated from the observed pH drop and growth data. The change in proton concentration during ammonium uptake increased from 0.015 to 0.035 mol H^+^/mol N-NH_4_^+^ as the nitrogen provided from ammonium increased from 6% to 9% when buffering is neglected (inset of Figure 3B). This large change in apparent ϕ/ψ (Equation 3) is influenced by the buffering capacity of the media including the bicarbonate equilibrium (Equation 4). The buffering of the algal cultures is shown to change dramatically throughout a batch culture as illustrated in Additional file 3: Figure S1.

This substantial change in the buffering capacity of the media during growth precludes accurate assessments of proton secretion (ϕ) from pH measurements and will be deferred to future studies with instrumentation designed for monitoring the proton balance more accurately. Nonetheless, it is clear that the proton imbalance must be considered in the overall mass balance. The role of carbonate buffering at higher pH is also evident as the same final pH was achieved in all cultures. More CO_2_ can absorb into the culture at higher pH as a result of the CO_2_↔HCO_3_^-^↔CO_3_^2-^ ‘carbonate’ equilibrium. This represents an additional bioreactor design constraint because the bicarbonate buffering not only masks changes in the proton concentration due to nitrogen metabolism, but also alters pH as a function of bioreactor CO_2_ transport rates and biological uptake rates. It is important to note that proton efflux in stoichiometric terms is very different from the simplistic local charge balance of 1:1 molar exchange for a transporter. Proton exchange per mole of nitrogen assimilated (ϕ/ψ) reflects the incorporation of hydrogen into biomass and allows for net charge balance by alternative cations. Understanding the combined role of nitrogen stoichiometry and CO_2_ dynamics is an important step towards implementing media-based control of pH that is needed for a large-scale algal process that does not rely on buffering and is a prerequisite to accurately closing the mass balance on algal biomass growth.

### Differential cellular regulation of nitrogen metabolism facilitates novel approach to pH control in microalgae

**Figure 4:**
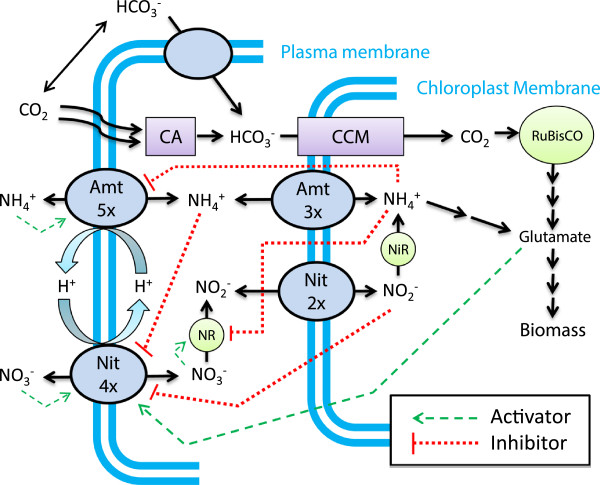
**Schematic of cellular regulation of nitrogen assimilation in the algae *Chlamydomonas reinhardtii*.** The number of identified transporters is in indicated on the respective membrane (i.e. 3x = three ammonium transporters on the chloroplast membrane). Dashed lines in the figure legend represent the regulatory elements involved in nitrogen metabolism. Additional key notations: Amt = ammonium transporter, Nit = nitrate transporter, NR = nitrate reductase, NiR = nitrite reductase, CA = carbonic anhydrase, CCM = carbon concentration mechanisms.

The pH instability observed in microalgal culture from preferential utilization of ammonium can be explained by cellular regulation of nitrogen assimilation. Ammonium has the ability to inhibit both nitrate transport and reduction as demonstrated in the bio-molecular model in Figure 4 generated from a literature review on nitrogen metabolism [6,30,31,33-37]. Upon depletion of ammonium, repression of the nitrate assimilatory pathway is alleviated and nitrate transport into the cell can occur, followed by a 2-step reduction to ammonium. The carbon concentration mechanism (CCM) allows accumulation of CO_2_ for RuBisCO and includes regulatory genes that are involved in functionality of both nitrogen assimilation pathways. Elevated CO_2_ gas-phase supplementation results in sufficient internal carbon for biomass formation and the CCM does not interfere with nitrogen metabolism.

**Figure 5:**
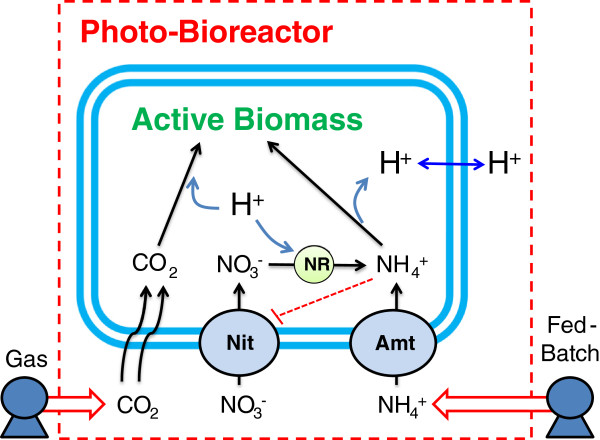
**Behavioral model of nitrogen utilization which facilities photobioreactor pH control.** This schematic represents the control elements that give rise to the observed pH response that can be controlled with incremental addition of ammonium. The presence of ammonium inhibits transport of nitrate into the algal cell and results in a net efflux of protons to the culture media. Following depletion of ammonium from the media, nitrate is transported into the cell and reduced to ammonium prior to assimilation, resulting in a net influx of protons from the culture media. Notations: Amt = ammonium transporter, Nit = nitrate transporter, NR = nitrate reductase.

The pH response to ammonium and nitrate utilization suggests that pH might be maintained if the addition of nitrogen salts exploited the intracellular regulatory mechanisms on nitrogen metabolism. This approach would require that cells be able to rapidly change from nitrate to ammonium assimilation and is surprisingly simple considering the complexity of nitrogen assimilation. The dramatically simplified control elements that give rise to the observed pH changes and facilitate pH control through alternating utilization of ammonium and nitrate are presented in Figure 5. When ammonium is present, nitrate assimilation is inhibited (1) and excess hydrogen is excreted from the cells as not all hydrogen from ammonium is required for biomass formation. Upon depletion of ammonium, nitrate assimilation can occur (2), which requires a net influx of hydrogen into the cell for reduction. The success of the control strategy relies on manipulating the direction of proton flux by alternating nitrogen sources to minimize the net effect on culture pH. In simple terms, algae are behaving as if they have uncontrolled consumption of ammonium which can be manipulated through fed-batch addition.

To test this behavior, a photoautotrophic culture of *Chlamydomonas reinhardtii* actively growing on nitrate was subjected to a pulse ammonium feed and displayed rapid pH drop (See Additional file 4: Figure S2). This suggested that there is sufficient expression of ammonium transporters during nitrate utilization so that the incremental addition of ammonium can be used for periodic reduction of pH [35]. This approach will thereby provide a means to implement the proposed stoichiometrically-balanced nitrogen feed (36 % N-NH_4_^+^and 64 % N-NO_3_^-^).

### Incremental addition of stoichiometrically-balanced media provides favorable pH for sustained growth of *Chlorella vulgaris*

**Figure 6:**
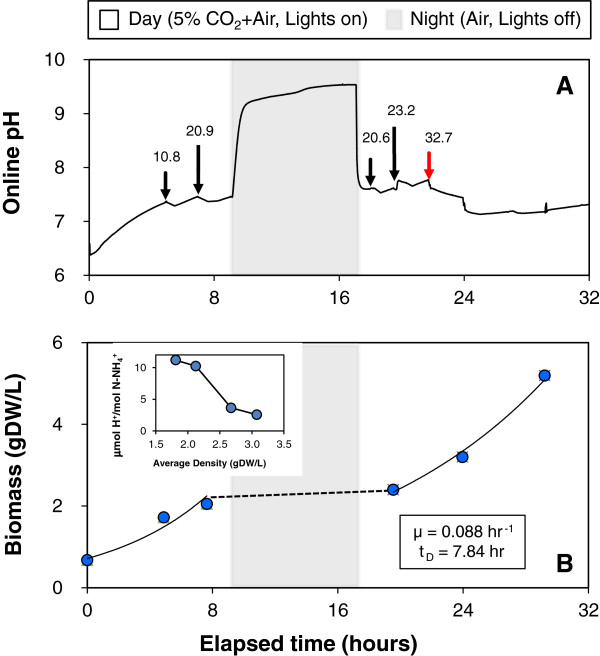
**Fed-batch addition of nitrogen for pH control in *Chlorella vulgaris*.** A photoautotrophic *Chlorella vulgaris* culture was grown in a trickle film photobioreactor on balanced growth media (0.3 gN/L at 36%N-NH_4_^+^ and balance as nitrate) under 5% CO_2_ (v/v) in air and subjected to a diurnal light cycle with a 16-hr photoperiod. The culture was initially grown on potassium nitrate with five ammonium nitrate additions made over the course of the growth period. Online pH was monitored continuously and averaged every minute to demonstrate pH fluctuations due to the ammonium nitrate additions indicated by the arrows and the concentration of ammonium in the reactor following each feeding (**A**). The red arrow signifies the unexpected pH response following the fifth ammonium nitrate addition. The optical density was measured at 550-nm to monitor biomass growth and converted to culture density using the biological conversion factor of 0.53 gDW/L/OD_550_ determined experimentally (**B**). The inset of (**B**) shows the relative change in proton concentration during ammonium metabolism following the first four NH_4_NO_3_ additions.

Fed-batch ammonium and nitrate addition was successfully implemented for growth of *Chlorella vulgaris* in an ultra-low path length trickle film photobioreactor (Figure 6). This reactor configuration was used to avoid the effect of light attenuation due to self-shading by minimizing film thickness and maximizing light penetration into the culture [38]. It is also important to note that the design of the enclosed trickle-film bioreactor with humidified gas and heat removal by a heat exchanger were implemented to eliminate evaporation so that observed increases in cell concentration would reflect growth and not evaporative concentration of the biomass. Potassium nitrate was provided at the beginning of the culture followed by ammonium nitrate additions after 6 hours of initial growth to ensure non-limiting nitrogen levels in the media. The first addition provided 10.8 mgN-NH_4_^+^/L and corresponded to 3.6% of the total 0.3 gN/L supplied over the reactor run. Subsequent NH_4_NO_3_ additions were increased to 32.7 mgN-NH_4_^+^/L without dropping the pH to inhibitory levels.

During the lighted hours, the pH was maintained between 7.0 and 7.8 as shown in Figure 6A. Within this pH range, bicarbonate is the dominant dissolved inorganic carbon species, which is believed to be the preferred carbon source for photosynthesis in *Chlorella vulgaris*[14,39]. After the first four ammonium nitrate additions, a pH drop was followed by a pH rise as ammonium was preferentially consumed followed by nitrate assimilation. It is interesting to note that the pH response following the last feeding (red arrow in Figure 6A) did not display the characteristic recovery of pH as nitrate levels reached 0.10 gNO_3_^-^/L (1.7 mM N), which would still be within the range of the low affinity transport system that operates above 1.1 mM N [40]. This unexpected response to ammonium addition suggests that other cellular regulation might be encountered that affect the pH control strategy.

The rapid rise in pH that occurs during the dark culture hours resulted because the supplemental CO_2_ was turned off ‘at night’ and the inorganic carbon species shift back to equilibrium with ambient CO_2_ (0.039%). Note that the mean gas residence time within the enclosed trickle film reactor bag enclosure was estimated at 6-min so that it takes about half an hour to change the gas composition. This dark period increase in pH illustrates the significant effect of CO_2_ transport and the resulting bicarbonate buffering system on the culture pH that result from 5% CO_2_ gas-phase supplementation. A long-term goal of our research program is to achieve high-density growth without CO_2_ buffering, which will require an understanding of the relationship between nitrogen regulation, carbon availability, and pH dynamics.

The accumulation of algal biomass during the 32-hr photobioreactor run with fed-batch nitrogen addition is shown in Figure 6B. This trickle film bioreactor run reached 5 gDW/L, which was a substantially higher density than observed in prior batch experiments with comparable feeding (0.3 gN/L). A possible explanation for the improved biomass yield is that maintenance of a more uniform pH during growth allowed the cells to more effectively utilize the energy available in the reduced nitrogen source. During the 24 hours of lighting, the culture grew at a specific growth rate of 0.088/hr (doubling time = 7.88 hr). The observed exponential growth suggests that the culture had not become limited by light, CO_2_ transport or inorganic nutrients during this period. The change in proton secretion during ammonium uptake (ϕ/*ψ*) after an ammonium nitrate pulse was smaller at higher cell densities (Figure 6B inset), which is consistent with the previously observed increase in buffering capacity at higher culture density (Additional file 3: Figure S1).

### pH Control based on nitrogen feed can be implemented during nitrogen-limited growth of *Chlamydomonas reinhardtii*

**Figure 7:**
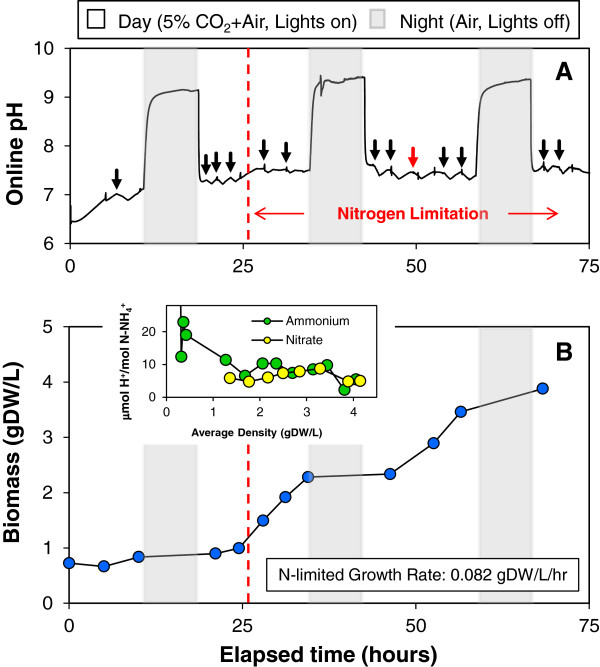
**pH control maintained during nitrogen limitation in *Chlamydomonas reinhardtii*.** A photoautotrophic *Chlamydomonas reinhardtii* culture was grown on balanced growth media under 5% CO_2_ (v/v) in air in a trickle film bioreactor and subjected to a diurnal light cycle with a 16-hr photoperiod. The culture was initially started on potassium nitrate until ammonium nitrate additions began after 10 hours of growth with a total of 0.6 gN/L added over the 75-hr growth period. The pH was measured online to monitor the proton imbalance from nitrogen metabolism before and after nitrogen depletion (indicated by red line at ~25 hrs) with black arrows indicating the time of each NH_4_NO_3_ addition, and the red arrow indicating the addition of NH_4_NO_3_ supplemented with a balance of non-nitrogen nutrient salts (**A**). The optical density was measured at 550-nm and converted to biomass density using the conversion factor of 0.51 gDW/L/OD_550_ determined experimentally (**B**). Also shown in (**B** inset) is the change in proton concentration per utilized nitrate.

Since nitrogen limitation is viewed as an important strategy to induce lipid accumulation in algae [41-43], an experiment was carried out with *Chlamydomonas* under a N-limited feed rate. The culture was grown in a trickle film photobioreactor to avoid light-limited growth and 5% CO_2_ (v/v) in air to provide excess carbon. The culture was initially grown on potassium nitrate with ammonium nitrate additions started after 10 hours of growth. The total base nitrogen level was doubled to 0.6 gN/L while retaining the balanced 36% nitrogen from ammonium and the balance as nitrate. A nutrient feeding strategy was used to initially maintain excess nitrogen, followed by growth where nitrogen became depleted between additions. The pH response to NH_4_NO_3_ addition was the same before and after depletion of excess nitrate, displaying the characteristic decline during NH_4_^+^ assimilation and recovery during subsequent NO_3_^-^ uptake. During the 16-hour lighted photoperiod of Days 1–4, the photobioreactor pH was maintained between 7.0 and 7.5 as shown in Figure 7A. Nightly pH swings were observed due to the removal of supplemental CO_2_ at night. During nitrogen limitation, linear growth was observed at a rate of 0.082 gDW/L/hr as shown in Figure 7B. The biomass yield during nitrogen-limited growth was determined to be 10.3 gDW/gN corresponding to the nominal biomass composition of 10% nitrogen by weight.

Under nitrogen-depleted conditions, the pH dropped, recovered and became constant as shown in more detail in Additional file 5: Figure S3. This suggests a nearly balanced proton secretion and uptake (ϕ/*ψ*) during the respective phases of ammonium and nitrate assimilation as illustrated by the Figure 7B inset. The observed change in pH with nearly identical magnitude but with opposite signs as ammonium and nitrate are consumed following NH_4_NO_3_ addition is consistent with charge balance of proton flux during nitrogen ion uptake. This simplistic view of pH change could greatly simplify pH control and might in part result from small rapid nutrient additions as well as buffering. However, it must be remembered that the CO_2_ and cell density-dependent buffering are significantly contributing to this pH response and will require more detailed study where buffering is minimized. Nonetheless, these short-term pH responses must be superimposed on the longer time-scale mass balance where the final redox state of nitrogen within the cell as well as the overall cation/anion uptake must be satisfied. Our near-term goals are to incorporate these models into an adaptive control strategy that will incorporate more comprehensive modeling of growth and pH dynamics, as it is dependent on the variable growth conditions that algae will experience in outdoor environmental conditions. Towards achieving this goal, a final experiment is presented for growth conditions under ambient (air) CO_2_ growth conditions.

### Carbon limitations reveal additional regulatory mechanisms on nitrogen metabolism in *Chlamydomonas* that lead to unpredictable pH dynamics

**Figure 8:**
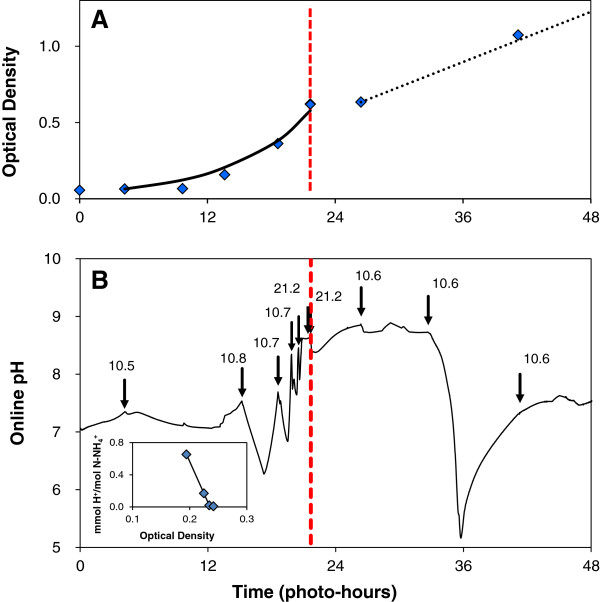
**Air-grown cultures demonstrate pH control through stoichiometry-balanced nitrogen feed prior to carbon limitation.** A photoautotrophic *Chlamydomonas reinhardtii* culture was grown on balanced growth media under 5% CO_2_ (v/v) in baffled shake flasks and subjected to a diurnal light cycle with a 16-hr photoperiod. The culture was started on potassium nitrate followed by ammonium nitrate additions (indicated by arrows and given in mgN-NH_4_^+^/L) began after 5-hrs of growth with a total of 0.3 gN/L added over the 48-hr growth period. The culture was allowed to grow past the onset of carbon limitation as indicated by the dotted red line (~21 hrs). The optical density was measured at 550-nm to monitor culture growth (**A**). The pH was measured online to monitor the proton imbalance from nitrogen metabolism (**B**). The inset of (**B**) shows the change in proton concentration per ammonium assimilated for the second to fifth NH_4_NO_3_ additions prior to carbon limitation.

To reveal cellular level metabolic controls on nitrogen assimilation in the absence of excess CO_2_, *Chlamydomonas reinhardtii* cultures were grown on air. This allowed testing of pH control using ammonium feed under conditions with minimal carbonate buffering. In addition, algal growth performance under reduced CO_2_ availability is particularly important for the goal of achieving a high CO_2_ utilization yield in commercial-scale photobioreactors. This study was conducted in gyratory shake flasks where the mass transfer rate (k_L_a) could be accurately measured to verify the onset of carbon limitation as the cause for linear growth. The transition to carbon limitation was imposed by providing a CO_2_ transport rate from air, which eventually became lower than the biological uptake rate of CO_2_ during growth on 0.3 gN/L (Figure 8A). Prior to carbon limitation, the culture grew at an exponential growth rate of μ = 0.17/hr (doubling time = 4.1-hr). Following carbon limitation, growth continued at a linear rate of 0.0274 OD_550_/hr (0.014 gDW/L/hr), which compares well with the predicted growth rate of 0.0307 OD_550_/hr (0.0160 gDW/L/hr) calculated based on 48.1% C by mass in the biomass and a CO_2_ transfer rate of 28.2 mg CO_2_/hr for ${\left(k_{L}a\right)}_{{\mathrm{CO}}_{2}}=48/\mathrm{hr}$[28]. These growth patterns support the intended experimental conditions of carbon-excess early in culture followed by carbon-limited growth after 29 photo-hours.

The pH was maintained between 6 and 8.5 during exponential growth (0–29 hours) with the expected pH decline and recovery following each NH_4_NO_3_ media addition that exhibited a more rapid response as the culture density increased (Figure 8B). The same regulatory mechanisms for nitrogen metabolism previously presented in Figures 5 and 6 are apparent in air-grown cultures as long as the carbon concentrating mechanism can maintain excess intracellular carbon. The inset of Figure 8B shows the decline in apparent pH change for growth on ammonium (ϕ/ψ) as anticipated, justifying the increase in NH_4_NO_3_ dosage at higher densities prior to carbon limitation. Following carbon limitation the pH dynamics became unpredictable and the pH ranged from 5 to 9. When aqueous carbon is limiting, the carbon concentration mechanism (CCM) can no longer provide sufficient CO_2_ to RuBisCO to support its maximum turnover rate. Feedback regulation from CCM to the nitrogen assimilation pathways acts to avoid intercellular accumulation of exogenously supplied or nitrate-derived ammonium and coordinate with 2-oxogluatarate availability for glutamate formation [33,44]. This altered nitrogen assimilation under carbon limitation adds complexity to the pH control strategy because the presence of ammonium is no longer sufficient to completely inhibit nitrate uptake [45]. A more detailed assessment of metabolic fluxes will clearly be required beyond simple on/off control that is possible when carbon is in excess. There is a tremendous value in further understanding carbon limited nitrogen assimilation due to its implications in both natural and engineered algal growth systems.

### Regulation of nitrogen assimilation in algae has important implications to achieving stoichiometrically-balanced media and associated control of culture pH

Although the concept of achieving pH control through stoichiometrically-balanced ammonium and nitrate is shown to have validity, this cannot be implemented with a simple batch media formulation. The preferential uptake of ammonium ions over nitrate results in a pH drop proportional to the ammonium provided. Our observation of preferential uptake of ammonium for a wide range of algae and cyanobacteria is consistent with the literature [46]. The implication of pH-independent preferential ammonium uptake is that algae will literally kill themselves by consuming ammonium ions even if nitrate is available to prevent media acidification. This result is in contrast to higher plants, which have the ability to utilize nitrate and ammonia in a balanced matter so that pH does not reach toxic levels. This is very notably observed in plant cell suspension culture where cultures can be grown to extremely high densities (> 50 gDW/L) on a mixture of ammonium and nitrate [47].

Physiologically it makes sense that cells within tissue must be capable of controlled metabolism of nitrogen so that the pH is maintained. In contrast, unicellular algae would not typically be grown at sufficiently high concentrations where metabolism would dramatically impact the surrounding water. In addition, ammonium ions are typically far less abundant in an environmental context (in part due to their favorable energetic utility). As a result, a high-density algal photobioreactor system creates an unnatural environment that microalgae have not evolved to accommodate and the metabolic control that does exist is problematic. The incremental addition of a fed-batch strategy for pH control is only a viable solution if the response to media addition is predictable. Current efforts are combining models of CO_2_ transport, CO_2_ equilibrium, and nitrogen assimilation as the basis of an adaptive control strategy for algal photobioreactors. Part of this experimentation seeks to explicitly evaluate the proton balance through measurement of ϕ_i_ (Equation 3) and allow for more explicit validation of the stoichiometrically-balanced media formulation approach presented in this research. A particularly important extension of this work is to confirm that pH control can be achieved for cyanobacteria since they are a platform for genetic engineering of biochemicals. Cyanobacteria have far more simplistic and less redundant NH_4_/NO_3_ assimilation pathway which may complicate the dynamic response to mixed nitrogen media additions; nitrogen-fixing cyanobacteria present an additional challenge that may preclude this pH strategy all-together.

## Conclusions

The scope of this work relates to understanding algal nitrogen metabolism in the context of achieving pH control in high-density algal photobioreactors [48]. Although a balance of protons during culture growth has received little attention, it has profoundly affected the development of algal media and growth conditions by selection of elevated CO_2_ and nitrate or urea, which avoid this problem. In terms of utilizing algal culture at a commercial outdoor scale of thousands of acres, pH control must be achieved for a widely dispersed culture that experiences highly variable day-to-day growth conditions. A balanced media to achieve pH control is a scalable alternative to expensive buffering, feedback acid/base control, or CO_2_ enrichment (which severely limits CO_2_ conversion efficiency). These observations are particularly important for proposed life cycle analyses that presume the ability to utilize wastewater in which ammonium is a dominant form of nitrogen. The work is also relevant to natural systems that experience agricultural runoff.

Several conclusions from the work presented here are a step toward the goal of controlling the pH in culture:

1) An analysis of basic photosynthetic reactions strongly suggests that current algal growth media are not stoichiometrically-balanced.

2) The benefit of elevated CO_2_ for algal growth is likely as much for pH control as it is for enhanced CO_2_ availability.

3) Media which contains ~36% of the total nitrogen in the form of ammonium ions is close to achieving a stoichiometric balance, which would avoid excess proton secretion or uptake.

4) If a stoichiometrically-balanced media is provided in batch culture, the preferential uptake of ammonium ions will result in a drop of pH to inhibitory/lethal levels.

5) Incremental addition of ammonium and nitrate ions can be used to control pH as long as the carbon availability is not severely limited and a substantial improvement in biomass yield on nitrogen can be observed.

6) The switch to preferential use of ammonium ions will take place in excess nitrate, or nitrogen-limited culture conditions.

7) Achieving pH control through metabolic use of oxidized and reduced nitrogen sources in large scale photobioreactors will require models of CO_2_ transport, CO_2_ equilibrium and nitrogen assimilation.

These observations of nitrogen assimilation in microalgae appear to be very general and make sense in terms of the physiology and environmental conditions under which these organisms typically grow; therefore, the approach to achieving pH control is anticipated to be true of both monocultures and natural algal consortia. The potential influence of microbial consortia within a non-aseptic algal culture system is an additional consideration that requires further study.

## Methods

### Algal culture

The algal strain *Chlorella vulgaris* was obtained from the UTEX culture collection (#2714) and algal strain *Chlamydomonas reinhardtii* cc-1690 was obtained from the *Chlamydomonas* Resource Center (http://www.Chlamy.org).

### Algal media

The following is the basal balanced WFAM-3g growth medium which contains sufficient nitrogen to support 3 grams dry weight per liter (gDW/L) based on 10% nitrogen by mass (0.3 gN/L at 36%N-NH_4_^+^ and 64%N-NO_3_^-^): 0.6 gKNO_3_, 0.61 gNH_4_NO_3_, 1-mL phosphates solution, 1-mL micronutrient stock solution, 0.024 gFe-EDTA · 2H_2_O, 0.121 gMgSO_4_ · 7H_2_O, 0.0486 gMgCl_2_, and 0.132 gCaCl_2_ · 2H_2_O in 1-L Milli-Q water. The phosphate solution contained 115 gK_2_HPO_4_ and 44.9 gKH_2_PO_4_ in 1-L Milli-Q water with the pH adjusted to 6.8. The micronutrient stock solution contained 1.83 gH_3_BO_3_, 0.54 gMnCl_2_ · 4H_2_O, 0.066 gZnSO_4_ · 7H_2_O, 0.031 gNa_2_MoO_4_ · 2H_2_O, 0.030 gCoCl_2_ · 6H_2_O, and 0.0075 gCuSO_4_ · 5H_2_O in 1-L Milli-Q water.

### Growth measurement

Optical density (OD) was measured using cuvettes with 1-cm path length in a Beckman Coutler DU 520 spectrophotometer at a wavelength of 550-nm (OD_550_) to avoid pigment absorption and maximize light-scattering contribution, referenced with tap water [49]. To measure the dry weight (DW), 1-mL of well-mixed culture was added to a pre-tared 1.7-mL Eppendorf tube measured using an analytical balance to 0.00001 accuracy. The cells were pelleted in a microfuge (14,000 RPM, 10-min). The supernatant was removed without disturbing the pellet and the cells were rinsed. The Eppendorf tubes were stored in a −20°C freezer and transferred to a −80°C freezer for at least 30-min with lids open immediately before freeze drying. Samples were dried in a Labconco freeze dryer (−70C coil) run for 24–36 hours depending on the number of samples. Samples were re-measured to 0.00001 using an analytical balance to determine the final weight of the tube and cell pellet.

### pH measurement

Online pH was measured using Cole-Parmer pH electrodes with double-junction BNC connectors interfaced to a LI-COR LI-1400 datalogger. Offline pH samples were measured using a Metler Toledo SevenEasy pH Meter S20. Samples were degassed on a gyratory shaker for 45-min to ensure dissolved inorganic carbon was in equilibrium with air to minimize the variability in offline pH readings due to partial degassing of samples during transport between reactor and pH meter.

### Nitrate measurement by Ion selective electrode (ISE)

The Nico2000 Nitrate ISE (ELIT 8021) and liquid double junction reference electrode (ELIT 003) were used for offline measurement of exogenous nitrate. The ISE and reference electrode were pre-conditioned in 10 g NO_3_^-^/L standard for at least 30-min. The ion selective electrode was calibrated using three independently prepared NaNO_3_ standards at 10 gNO_3_^-^/L each serially diluted to 0.01 g/L. The ISE and reference electrode were left in the experimental samples until the electrical potential remained constant for 1-min. Due to the drift in electrical potential that occurred with extended use, the calibration standards and samples were measured the same day. Nitrogen-free media was used to determine the background contribution from interfering ions to adjust the baseline concentration down to 0 gNO_3_^-^/L. Fresh media was used as the positive control for each treatment.

### Light cycle and temperature

All batch and fed-batch experiments were executed in a Conviron BDW120 walk-in incubator. High intensity lighting was supplied to cultures using Philips 400 W high-pressure sodium vapor and Philips 400 W metal-halide lamps. These lights were set to diurnal cycle on an 8-hr dark/16-hr light cycle to imitate sunlight. In the first and last hour of the photoperiod, the light intensity was stepped to 1/3 of the maximum; combined with lamp warm-up, this avoided morning photo-inhibition. The temperature within the incubator was maintained at 28°C during the day and dropped to 25°C during the dark hours – ramped linearly over a 1 hour period.

### Shake flasks

Cultures for batch experiments and inocula for the photobioreactors were grown in 500-mL shake flasks with 75-mL, and gas supplementation at 5% CO_2_ (v/v) in air at 120 RPM on a New Brunswick Scientific G-10 gyratory platform shaker. Each flask was sealed with a silicone stopper with inlet and outlet gas lines and connected in series through these gas lines. For each flask, a 0.2-μM Millipore filter was used on the inlet line and the outlet line was loosely plugged with cotton. The mass transfer coefficient was determined by the unsteady state sulfite addition method for oxygen mass transfer using 0.61-M Na_2_SO_3_ to react out dissolved O_2_ catalyzed by copper (II) sulfate (0.1-M). The measured mass transfer coefficient for oxygen was then correlated to CO_2_ mass transfer using the relative diffusivities (D) in water, given by the relationship:

$$\;{\left(k_{L}a\right)}_{CO_{2}}={\left(k_{L}a\right)}_{O_{2}}\sqrt{\frac{D_{CO_{2}}}{D_{O_{2}}}}.$$

### Preparation of reactor inocula

Cultures were grown in media with 0 or 4.5% nitrogen as ammonium at 0.3 gN/L and all non-nitrogen components consistent with the stoichiometric growth media under 5% CO_2_ (v/v) supplementation in shake flasks. Cells were harvested by centrifugation (2000-g for 5-min at 24°C ), washed in nitrogen-free media to remove extracellular nitrogen, centrifuged a second time, and re-suspended in nitrogen-free media.

### Loop air-lift photobioreactor

The air-lift bioreactors with working volume of 1.5-L were constructed from translucent polyethylene plastic tubing using a W-605A 24-inch Single Impulse heat-sealer with 5 mm seal (Recycle = 1, Congealing = 4, Sealing = 4) to form the bag configuration. Details of the reactor dimensions and pictures are described elsewhere (Tuerk, 2011), which is available online [38]. A ceramic sparger attached to plastic tubing was inserted into the reactor through a hole near the top of the bag to form the riser in the narrow side. A hole cut above the liquid level served as the inoculation and sample port. A Cole-Parmer pH electrode was inserted into the bag reactor in the airlift ‘downcomer’. The bag reactor was placed between two metal wire racks without blocking light to limit the thickness to approximately 0.75-in. Gas was sparged into the reactor at 0.31 VVM at 5% CO_2_ (v/v) in air. The average light flux to the culture was 252 μmol/m^2^/s over a total surface area of 0.11 m^2^, and was determined by holding a LI-COR PI-190 quantum (PAR) light sensor normal to the bag surface.

### Trickle film photobioreactor

The trickle film photobioreactor with working volume of 500-mL was the basis of extensive algal photobioreactor development studies described elsewhere [38]. The specific configuration used in the work reported here consisted of two screens (fiberglass window screen stock) enclosed in a clear plastic bag (2 mil 30-in. × 34-in.) filled with humidified gas at 5% CO_2_ (v/v) in air. A 1-L glass reservoir was sealed with a silicone stopper that was fitted with a gas port, the liquid return, sample port, and temperature probe. The culture was collected in this reservoir after flowing down the screen and pumped out the bottom through a sidearm using a Watson-Marlow peristaltic pump 601S at 0.5 to 1-L/min. The culture temperature was maintained at 25°C through a heat exchanger in the culture recycle loop. The average measured light flux to the screen was 282 μmol/m^2^/s with a screen area of 0.3 m^2^, and was measured by holding the light sensor normal to the surface of the screen within the bag enclosure.

## Competing interests

WRC is the president of a biotechnology startup company Calyx Bioprocessing, LLC that was established to help support bioprocessing commercialization and consulting efforts. A patent has been filed for the described trickle film photobioreactor that was released by Penn State Research Corporation to WRC to facilitate commercialization.

## Authors’ contributions

MLS designed, executed and analyzed the batch and fed-batch experiments, and developed the ion selective electrode procedure. WRC developed the balanced growth media, conceived the pH control strategy, and designed the trickle-film photobioreactor as well as oversaw experiment design, execution and analysis. Both authors drafted, read and approved the final manuscript.

## Supplementary Material

Additional file 1 **Algebraic expressions for stoichiometric coefficients for various nitrogen source.** The stoichiometric coefficients were evaluated for various nitrogen sources for photoautotrophic growth in terms of biomass composition. Differences illustrate the fundamental inconsistency if proton imbalance is not considered. Click here for file

Additional file 2 **Calculation of ammonium content in stoichiometrically-balanced media for photosynthetic algal growth.** The approach for determining the amount of nitrogen in the form of ammonium (Δ) to be contained in our photoautotrophic growth media is presented. Click here for file

Additional file 3: Figure S1 Increased buffering capacity of algal cell cultures during batch growth. Titrations were performed to determine changes in culture buffering capacity of the culture as it grows which includes the combined effects of increased cell density and media exhaustion. Click here for file

Additional file 4: Figure S2 Inhibition of nitrate assimilation by ammonium in *Chlamydomonas reinhardtii.* Experimental results demonstrated a switch to ammonium metabolism upon its addition to a photoautotrophic *Chlamydomonas reinhardtii* actively growing on nitrate with minimal lag time between alternating nitrogen metabolism. Click here for file

Additional file 5: Figure S3 Detailed pH response of *Chlamydomonas reinhardtii* to ammonium nitrate addition under nitrogen-excess and nitrogen-limited growth conditions. The pH response to ammonium addition was the same before and after the depletion of excess media nitrate. Nitrogen depletion from the media corresponded to a constant experimental pH between ammonium nitrate additions. Click here for file
