# Supplementary material for: Achieving pH control in microalgal cultures through fed-batch addition of stoichiometrically-balanced growth media
Source: BMC Biotechnol. 2013 May 7;13:39. doi: 10.1186/1472-6750-13-39 (PMC3751429; doi:10.1186/1472-6750-13-39)
Supplement: Additional file 1 — Algebraic expressions for stoichiometric coefficients for various nitrogen source. The stoichiometric coefficients were evaluated for various nitrogen sources for photoautotrophic growth in terms of biomass composition. Differences illustrate the fundamental inconsistency if proton imbalance is not considered. [file 1472-6750-13-39-S1.pdf]

Additional File 1: Algebraic expressions for stoichiometric coefficients for photosynthetic growth on various nitrogen sources

The stoichiometric coefficients were evaluated for various nitrogen sources for photoautotrophic growth in terms of the biomass composition (x, y, z) when extracellular metabolites and proton balance are not considered.

$$\alpha_i CO_2 + \psi_i \mathbb{N}_i + \delta_i H_2O \overset{hv}{\rightarrow} CH_x N_y O_z + \lambda_i O_2$$

Nitrogen Source	Stoichiometric Coefficients			
	**α**	**ψ**	**δ**	**λ**
N_2	1	$\frac{y}{2}$	$\frac{x}{2}$	$1+\frac{x}{4}-\frac{z}{2}$
NH_3	1	y	$\frac{x}{2}-\frac{3y}{2}$	$1+\frac{x}{4}-\frac{3y}{4}-\frac{z}{2}$
NH_4^+	1	y	$\frac{x}{2}-2y$	$1+\frac{x}{4}-y-\frac{z}{2}$
NO_3^-	1	y	$\frac{x}{2}$	$1+\frac{x}{4}+\frac{3y}{2}-\frac{z}{2}$
HNO_3	1	y	$\frac{x}{2}-\frac{y}{2}$	$1+\frac{x}{4}+\frac{5y}{4}-\frac{z}{2}$
NH_4OH	1	y	$\frac{x}{2}-\frac{5y}{2}$	$1+\frac{x}{4}-\frac{3y}{4}-\frac{z}{2}$
$(NH_2)_2CO$ (urea)	$1-\frac{y}{2}$	$\frac{y}{2}$	$\frac{x}{2}-y$	$1+\frac{x}{4}-\frac{3y}{4}-\frac{z}{2}$
$(NH_4)_2CO_3$ (ammonium carbonate)	$1-\frac{y}{2}$	$\frac{y}{2}$	$\frac{x}{2}-2y$	$1+\frac{x}{4}-\frac{3y}{4}-\frac{z}{2}$
$(NH_4)HCO_3$ (ammonium bicarbonate)	$1-y$	y	$\frac{x}{2}-\frac{5y}{2}$	$1+\frac{x}{4}-\frac{3y}{4}-\frac{z}{2}$

Differences in stoichiometric coefficients illustrate the fundamental inconsistency of the overall mass balance if proton uptake and secretion are not considered.
