# Supplementary material for: Achieving pH control in microalgal cultures through fed-batch addition of stoichiometrically-balanced growth media
Source: BMC Biotechnol. 2013 May 7;13:39. doi: 10.1186/1472-6750-13-39 (PMC3751429; doi:10.1186/1472-6750-13-39)
Supplement: Additional file 2 — Calculation of ammonium content in stoichiometrically-balanced media for photosynthetic algal growth. The approach for determining the amount of nitrogen in the form of ammonium (Δ) to be contained in our photoautotrophic growth media is presented. [file 1472-6750-13-39-S2.pdf]

Additional File 2: Calculation of ammonium content in stoichiometrically balanced media for photosynthetic algal growth

From the photoautotrophic growth equation (Equation SF2.1), elemental balances can be written for carbon, hydrogen, nitrogen, and oxygen (Equations SF2.2A-D). The goal of the media development is to select the ammonium-nitrogen to total nitrogen molar ratio (Δ) where the pH is balanced; therefore, $\phi_{\Delta N}$ has been taken to be zero. For growth on a mixture of ammonium and nitrate salts:

$$\alpha CO_2 + \Delta\psi_{\Delta N} NH_4^+ + (1-\Delta)\psi_{\Delta N} NO_3^- + \delta H_2O \overset{hv}{\rightarrow} CH_xN_yO_z + \lambda O_2 + \phi_{\Delta N} H^+ \qquad \textbf{SF2.1}$$

Carbon: $$\alpha = 1 \qquad \textbf{SF2.2A}$$

Hydrogen: $$\delta = \frac{x}{2} - 2\Delta y \qquad \textbf{SF2.2B}$$

Nitrogen: $$\psi_{\Delta N} = y \qquad \textbf{SF2.2C}$$

Oxygen: $$\lambda = 1 + \frac{3y}{2} - \frac{5\Delta y}{2} + \frac{x}{4} - \frac{z}{2} \qquad \textbf{SF2.2D}$$

From the elemental balances written from Equation SF2.1, three unknown stoichiometric coefficients (δ, Δ, and λ) result with only two unspecified equations (hydrogen and oxygen balances). The desired parameter Δ can be determined if an additional constraint is introduced based on the experimentally observed pH-balanced growth on urea where there is minimal net extracellular exchange of protons. The elemental balances for growth on urea when ϕ_u=0 are given in Equations SF2.4A-D.

$$\alpha CO_2 + \psi_u CO(NH_2)_2 + \delta H_2O \overset{hv}{\rightarrow} CH_xN_yO_z + \lambda O_2 + \phi_u H^+ \qquad \textbf{SF2.3}$$

Carbon: $$\alpha + \Psi_u = 1\ ;\ \alpha = 1 - \frac{y}{2} \qquad \textbf{SF2.4A}$$

Hydrogen: $$\delta = \frac{x}{2} - y \qquad \textbf{SF2.4B}$$

Nitrogen: $$\psi_u = \frac{y}{2} \qquad \textbf{SF2.4C}$$

Oxygen: $$\lambda = 1 - \frac{3y}{4} + \frac{x}{4} - \frac{z}{2} \qquad \textbf{SF2.4D}$$

To replicate the balanced degree of reduction that is achieved for growth on urea, an energetic constraint can be defined to achieve no net flux of protons during growth on a

mixed nitrogen source of ammonium and nitrate. By settings the efficiency of CO_2 reduction relative to water splitting, $\left(\frac{\alpha}{\delta}\right)_{urea}$ equal to $\left(\frac{\alpha}{\delta}\right)_{(NH_4^+:NO_3^-)}$, Equation SF2.5 can be used to determine an expression for Δ in terms of only the biomass composition as shown in Equation SF2.6.

$$\left(\frac{\alpha}{\delta}\right)_{urea} = \frac{1-\frac{y}{2}}{\left[\frac{1}{2}(x-2y)\right]} = \left(\frac{\alpha}{\delta}\right)_{\Delta N} = \frac{1}{\left[\frac{1}{2}(x-4\Delta y)\right]} \qquad \textbf{SF2.5}$$

$$\Delta = \frac{(4-x)}{4(2-y)} \qquad \textbf{SF2.6}$$

Given a representative biomass composition, the fraction of nitrogen that should be present as ammonium (Δ) in pH-balanced photoautotrophic growth media can be calculated for $\phi_{\Delta N}$ =0.
