# Supplementary material for: Achieving pH control in microalgal cultures through fed-batch addition of stoichiometrically-balanced growth media
Source: BMC Biotechnol. 2013 May 7;13:39. doi: 10.1186/1472-6750-13-39 (PMC3751429; doi:10.1186/1472-6750-13-39)

Supplemental Figure 1: Increased buffering capacity of algal cell cultures during batch growth.

To assess the buffering capacity of media, algae cultures at different stages of increasing growth were titrated with HCl. Photoautotrophic *Chlorella vulgaris* cultures were grown on 0.3 gN/L in shake flasks with 5% (v/v) CO_2 (v/v) in air. The cultures were grown on KNO_3 0-9% N-NH_4^+ provided as NH_4OH. The cultures were degassed to ensure samples were in equilibrium with air prior to performing the titrations and the effects of the bicarbonate buffering system would be negligible. HCl (0.014N) was added to these total culture samples with the volume of addition measured using a burette. Fresh media (0.3 gN/L with NH_4NO_3 and KNO_3 at 36%N-NH_4^+) was included as a comparison to the buffering capacity when cells were present at varying densities. This study reflects the overall buffering capacity of the culture and does not distinguish between the contributions of increasing cell density and media exhaustion.

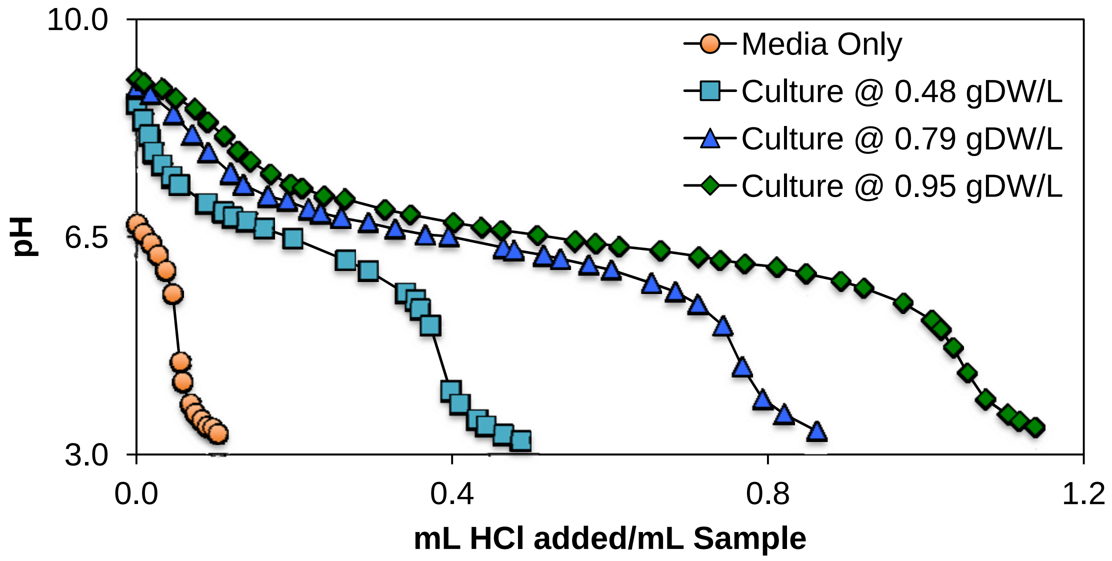

Supplement: Additional file 3: Figure S1 — Increased buffering capacity of algal cell cultures during batch growth. Titrations were performed to determine changes in culture buffering capacity of the culture as it grows which includes the combined effects of increased cell density and media exhaustion. [file 1472-6750-13-39-S3.pdf]
