# Supplementary material for: Achieving pH control in microalgal cultures through fed-batch addition of stoichiometrically-balanced growth media
Source: BMC Biotechnol. 2013 May 7;13:39. doi: 10.1186/1472-6750-13-39 (PMC3751429; doi:10.1186/1472-6750-13-39)

Supplemental Figure 2: Inhibition of nitrate assimilation by ammonium in *Chlamydomonas reinhardtii*

Photoautotrophic *Chlamydomonas reinhardtii* cultures were grown in 1.5-L loop air-lift photobioreactors under 5% CO_2 (v/v) in air. The control batch culture (squares) was grown on only KNO_3 at 0.3 gN/L. The experimental culture (circles) was initially grown on KNO_3 with NH_4Cl added after 12 photo-hours as indicated by the black arrow, such that 0.3 gN/L was provided over the growth period at 36% N-NH_4^+. The pH was monitored online and averaged every hour (A). The nitrate concentration in the media was measured using an ion selective electrode (B). The biomass for each culture was normalized by the initial amount of biomass to compare growth between cultures (C).

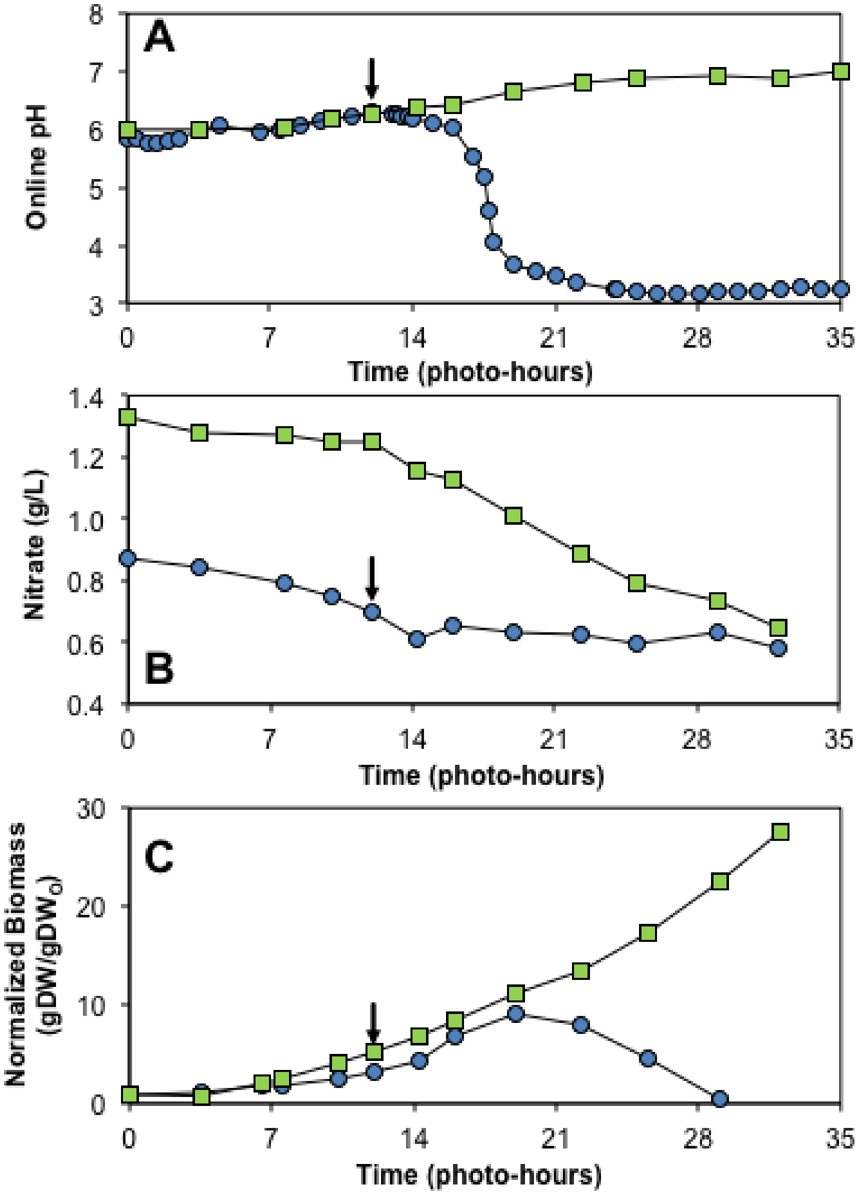

Supplement: Additional file 4: Figure S2 — Inhibition of nitrate assimilation by ammonium in Chlamydomonas reinhardtii. Experimental results demonstrated a switch to ammonium metabolism upon its addition to a photoautotrophic Chlamydomonas reinhardtii actively growing on nitrate with minimal lag time between alternating nitrogen metabolism. [file 1472-6750-13-39-S4.pdf]
