# Supplementary material for: Achieving pH control in microalgal cultures through fed-batch addition of stoichiometrically-balanced growth media
Source: BMC Biotechnol. 2013 May 7;13:39. doi: 10.1186/1472-6750-13-39 (PMC3751429; doi:10.1186/1472-6750-13-39)

Supplemental Figure 3: Detailed pH response of *Chlamydomonas reinhardtii* to ammonium nitrate addition under nitrogen-excess and nitrogen-limited growth conditions.

A photoautotrophic *Chlamydomonas reinhardtii* culture was grown on KNO_3 under 5% CO_2 (v/v) in air in a trickle film bioreactor and was subjected to a diurnal light cycle with a 16-hr photoperiod. The pH was measured online to monitor the proton imbalance from nitrogen metabolism before and after depletion (indicated by red line at ~8 hrs) with arrows indicating the time of each NH_4NO_3 addition and the amount given as mg N-NH_4^+/L (**A**). The nitrate concentration in the reactor was measured offline using an ion selective electrode (**B**).

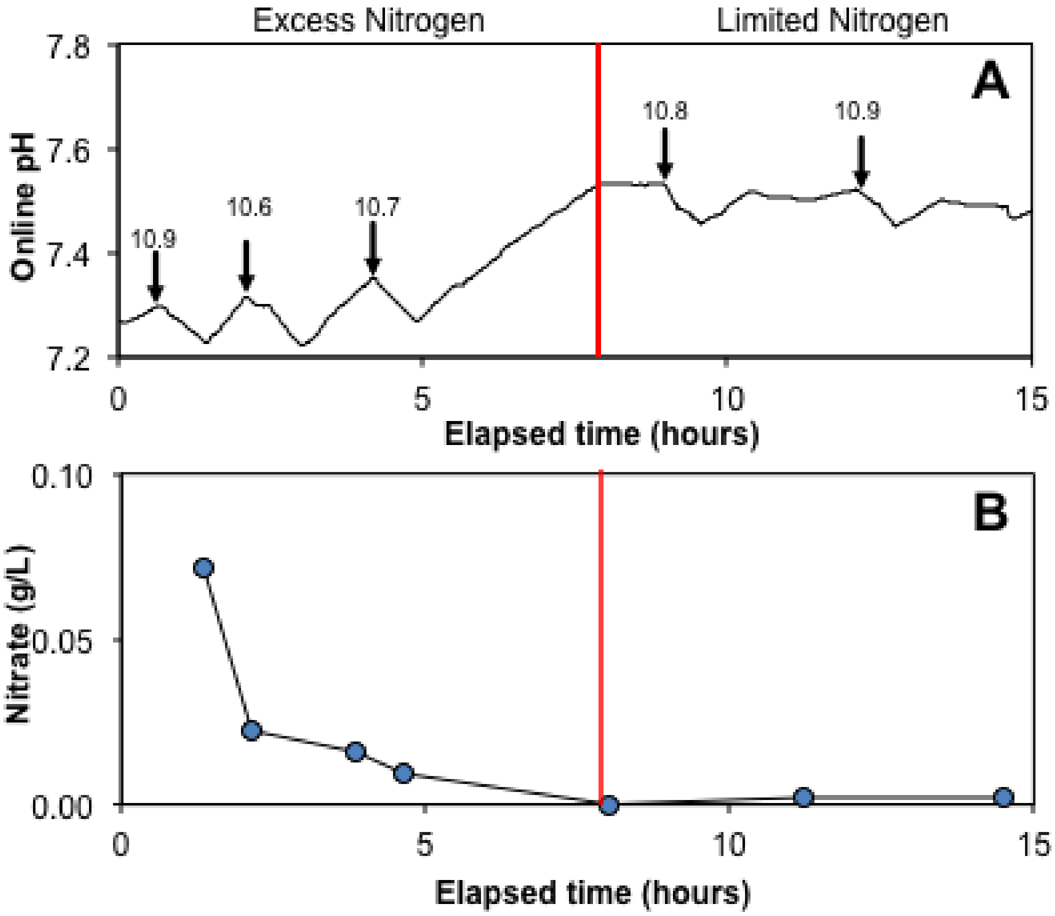

Supplement: Additional file 5: Figure S3 — Detailed pH response of Chlamydomonas reinhardtii to ammonium nitrate addition under nitrogen-excess and nitrogen-limited growth conditions. The pH response to ammonium addition was the same before and after the depletion of excess media nitrate. Nitrogen depletion from the media corresponded to a constant experimental pH between ammonium nitrate additions. [file 1472-6750-13-39-S5.pdf]
